# Statin Use and Cancer Incidence in Patients with Type 2 Diabetes Mellitus: A Network Meta-Analysis

**DOI:** 10.1155/2018/8620682

**Published:** 2018-09-04

**Authors:** Yi-Bing Hu, En-De Hu, Rong-Quan Fu

**Affiliations:** ^1^Department of Gastroenterology and Hepatology, Jinhua Municipal Central Hospital, Jinhua Hospital of Zhejiang University, Jinhua, 321000 Zhejiang, China; ^2^Department of Anesthesiology, Perioperative Medicine, The Second Affiliated Hospital and Yuying Children's Hospital of Wenzhou Medical University, Wenzhou, 325000 Zhejiang, China; ^3^Key Laboratory of Anesthesiology of Zhejiang Province, The Second Affiliated Hospital and Yuying Children's Hospital of Wenzhou Medical University, Wenzhou, 325000 Zhejiang, China; ^4^Department of Infection Diseases, The Third Affiliated Hospital of Wenzhou Medical University, Wenzhou, 325000 Zhejiang, China

## Abstract

**Background:**

Type 2 diabetes mellitus (T2DM) patients are involved closely with cancer. This work aims to conduct a systematic review and network meta-analysis (NMA) to examine the effect of different types of statins on cancer incidence in patients with T2DM.

**Methods:**

We systematically searched the Cochrane Library, PubMed, Embase, and Wanfang databases from January 1999 to March 2017. We performed a pairwise meta-analysis to estimate the pooled ratios (ORs) and 95% confidence intervals (CIs). A NMA was performed to compare different types of statins.

**Results:**

Seven publications were included. In pairwise meta-analysis, the incidence of cancer in T2DM patients was reduced when simvastatin, atorvastatin, pravastatin, fluvastatin, lovastatin, rosuvastatin, and pitavastatin were used. In the result of NMA, the usage of simvastatin (RR 0.30 and 95% CI 0.16–0.56), atorvastatin (RR 0.29 and 95% CI 0.09–0.88), pravastatin (RR 0.34 and 95% CI 0.12–0.93), fluvastatin (RR 0.27 and 95% CI 0.09–0.83), rosuvastatin (RR 0.22 and 95% CI 0.10–0.49), and pitavastatin (RR 0.33 and 95% CI 0.20–0.57) was superior to the nonstatin groups. When compared with six other statins, rosuvastatin appeared to be the best one.

**Conclusions:**

Different statins can reduce the risk of cancer in patients with T2DM. Our analyses suggest that rosuvastatin may be more effective than others.

## 1. Introduction

Diabetes mellitus (DM), mainly type 2 diabetes mellitus (T2DM), and cancer are two critical diseases which have a high incidence [1]. As reported by the National Central Cancer Registry of China, there were about 4,292,000 new cancer patients only in 2015 [2]. Previously, T2DM was regarded as a chronic disease only for the elder people, but recently its incidence was increased both in adolescents and children [3, 4]. Since 1959, evidence from many studies had revealed that there was an association between T2DM and cancer, and patients who had T2DM were more likely to be diagnosed with cancer than patients who had not [5–8]. Therefore, it is vital to find new strategies to reduce the occurrence of cancer for patients with T2DM.

By inhibiting 3-hydroxy-3-methylglutaryl coenzyme A (HMG-CoA) reductase, statins could reduce the levels of cholesterol. For decades, statins have been commonly used to treat the hypercholesterolemia [9–11]. Currently, statins were proved to play a role in decreasing the risk of depression, heart failure, Parkinson disease, and cerebrovascular disease [12–15].

A growing number of studies have suggested statin-related potential for reducing the risk of cancer [16–18]. A lot of evidence has also shown its beneficial effects in cancers, including prostate, breast, lung, and colorectal cancers [19–22]. Experimental results *in vitro* have suggested the effect of statins on growth, migration, apoptosis, and autophagy of cancer cells [23–25]. According to the data from *in vivo* studies, statins may act as a preventive drug for hepatocellular carcinoma, bladder cancer, and malignant glioma [26–28]. However, the role of statins on the incidence of cancer in patients with T2DM has not been well documented.

Given the high incidence of cancer in patients with T2DM and the widespread use of statins, it is necessary to understand the relationship between statins and cancer. For this purpose, we performed this meta-analysis to evaluate the impact of different types of statins on the risk of cancers with T2DM. Network meta-analysis (NMA) can provide strong evidence to support potential strategies for T2DM-related cancers for different statin subtypes.

## 2. Materials and Methods

### 2.1. Search Strategy

To identify the studies, we search the Cochrane Library, PubMed, Embase, Wanfang, and Chinese National Knowledge Infrastructure databases from January 1999 to March 2017 without any language restrictions. Search key terms included “statin(s),” “cancer,” and “diabetes mellitus.” The search was limited to human subjects. The references of published articles were manually reviewed to complement the search. The titles and abstracts were scanned to exclude the irrelevant studies, and the full texts of the remaining articles were carefully read to assess whether they had enough information. Unpublished articles were also excluded. The protocol for meta-analysis was conducted in accordance with preferred reporting items for systematic reviews and meta-analyses (PRISMA) [29].

### 2.2. Selection Criteria

The studies which were included in this meta-analysis should meet the following criteria: (1) the design of studies should be controlled strictly, including case-control studies, cohort studies, and RCTs; (2) no prior diagnosis of any cancer prior to the date of cancer diagnosis; (3) studies should specific one or more types of statins, including rosuvastatin, atorvastatin, simvastatin, pravastatin, fluvastatin, cerivastatin, and lovastatin; (4) studies should be designed to evaluate the impact of statins on the risk of cancers with T2DM; (5) studies should have provided sufficient information to calculate odds ratios (ORs) and 95% confidence intervals (CIs). If publications were duplicated or overlapped, only the most recent one was included. All the studies should be peer review papers in full length.

### 2.3. Data Extraction and Quality Assessment

Two investigators independently screened the titles and abstracts and reviewed the full paper. If there was any disagreement regarding data extraction, it would be resolved with a third review. When some information or data was needed but unclear, the original authors would be asked by email. The following characteristics were collected from each study: publication data (first author, year of publication, country/district), study design, population characteristics, type of statins, number of total sample size, and number of cancer cases. The quality of case-control studies was assessed using the Newcastle-Ottawa Quality Assessment Scales (NOS), and the quality of RCTs was evaluated by the Jadad scale [30, 31].

### 2.4. Data Analysis

To compare the risk of cancer in diabetic patients when they were exposed to different statins, two methods were used.

First, a traditional pairwise meta-analysis was performed to directly compare the same interventions. The pooled OR and 95% CI were calculated basing on the clear data described in the included articles. Considering the between-study heterogeneity, a quantity I [2] statistic was estimated to describe the percentage of overall variation. If the value was smaller than 50%, it suggested there was not an obvious heterogeneity; then, a fixed effects model was used. If not, a random effects model was chosen. Funnel graph and Egger et al. [32] regression asymmetry tests were used to evaluate publication bias.

Then, by using the NMA package of STATA 14.0, a NMA was presented. To compare the effect of different statins on diabetic patients for the risk of cancers directly and indirectly, ORs were calculated by employing frequentist techniques, which were performed in the form of graph. A network plot, inconsistency plot, predictive interval plot, comparison-adjusted funnel plot, and rank diagram of respective statin types were applied using NMA tool as described in published article [33].

## 3. Result

### 3.1. Description of Studies

**Figure 1:**
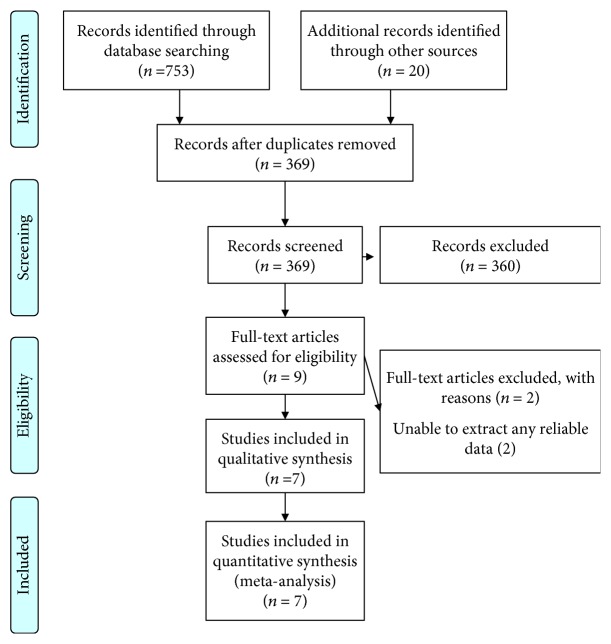
Flow chart of studies selected for this meta-analysis.

Figure 1 is a PRISMA flowchart depicting the electronic searching process for relevant studies. Briefly, 369 potential relevant studies were recovered through the combined initial database and reference searches. After reviewing their titles and abstracts, 360 articles were excluded. Then, by assessing the detailed of the remaining studies, two studies were excluded because of available data missing. Finally, seven studies reporting the effect of statins on type 2 diabetes mellitus patients were included in present analysis. Among the 7 studies enrolling a total of 23,555 participants, 2 studies are random control trials and 5 studies are case-control studies [34–40].  Table 1 shows the characteristics of study.

The quality of the two RCTs was assessed using the Jadad scale. According to the scores, both of them were high. The methodological quality of the remaining 5 studies was evaluated according to the NOS. If the scores of studies were 6 or more, they would be regarded as high quality. The scores of included studies are displayed in Table 1.

### 3.2. The Network Diagram of Eight Intervention Factors

**Figure 2:**
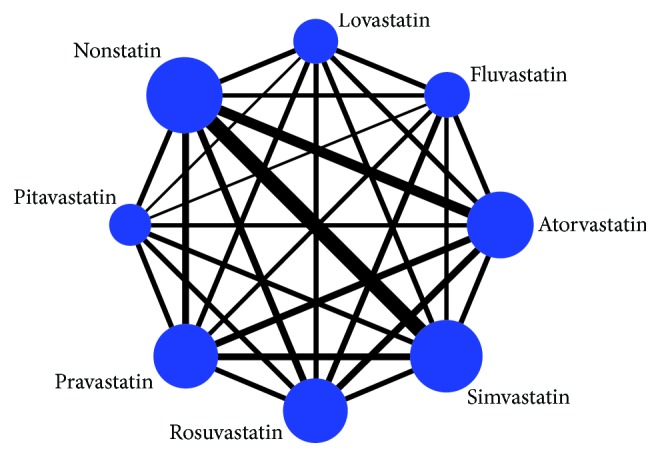
Network of comparisons for the multiple-treatment meta-analysis for cancer occurrence in diabetic patients. The width of the solid lines presents proportional to the number of trials that compares each pairwise treatment, and the size of each node presents proportional to the number of participants.

Figure 2 presents network diagrams for analyses of 8 interventions to cancer occurrence in diabetic patients. Different types of statins prevent the occurrence of cancer in diabetic patients, and the node of simvastatin is the largest, indicating that the more samples are included, whereas the pitavastatin sample is the least. The solid lines between the two nodes show the state of direct comparison, and there is direct evidence of the comparison in each state of the study, so there is a connection between the heads. Among all comparisons, the study of direct comparisons between observation and simvastatin or observation and atorvastatin is relatively more than others and can exert meta-analysis. However, most of the comparisons especially contain pitavastatin, lovastatin, and fluvastatin are too small to as direct evidence. To compare the effects of different statins on cancer occurrence in diabetic patients, we consider the use of network meta-analysis to analyze the relationship between the eight states.

### 3.3. Direct Meta-Analysis Results

**Figure 3:**
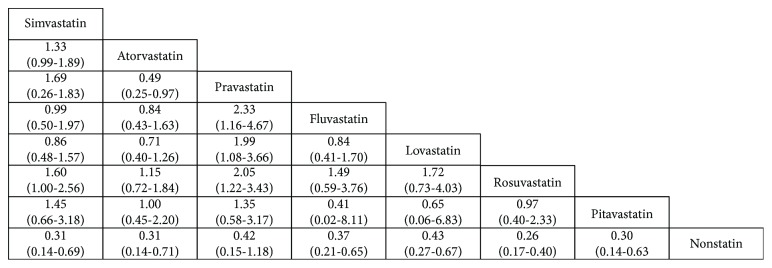
Direct meta-analysis results for pairwise comparisons were represented by ORs with 95% CI. ORs which were higher than 1 favored the column-defining treatment and ORs which were lower than 1 supported horizontal treatment. Statistically significant differences between interventions are shown in bold, underlined font. OR: odds ratio; CI: credibility interval.

As shown in Figure 3, 28 direct meta-analysis results for pairwise comparisons consisted by 8 interventions. For the diabetes patients with different types of statins or nonstatin, the occurrence of cancer had been different which were statistically significant or not statistically significant. When operating heterogeneity test, if the I square was greater than 50%, we selected the random effects model; if the I square was less than 50%, we selected the fixed effects model. The incidence of cancer in diabetic patients was reduced when statins were used, and their ORs (95% CI) were as follows: simvastatin (OR 0.31 and 95% CI 0.14–0.69), atorvastatin (OR 0.31 and 95% CI 0.14–0.71), pravastatin (OR 0.42 and 95% CI 0.15–1.18), fluvastatin (OR 0.37 and 95% CI 0.21–0.65), lovastatin (OR 0.43 and 95% CI 0.27–0.67), rosuvastatin (OR 0.26 and 95% CI 0.17–0.40), pitavastatin (OR 0.30 and 95% CI 0.14–0.63). The use of different statins in the prevention of cancer in diabetic patients was different. Rosuvastatin may be more effective than simvastatin (1.60 and 1.00–2.56) and pravastatin (2.05 and 1.22–3.43); however, pravastatin in the prevention of cancer in patients with diabetes was worse than lovastatin (1.99 and 1.08–3.66), fluvastatin (2.33 and 1.16–4.67), but better than atorvastatin (0.49 and 0.25–0.97).

### 3.4. Consistency Test

**Figure 4:**
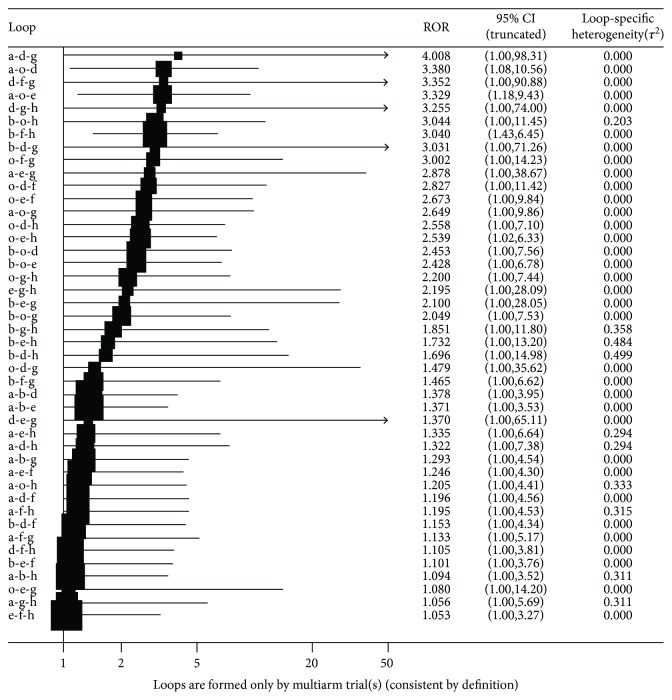
Consistency of direct evidence and indirect evidence was based on the value of ROR. The letters in the figure represented different treatment measures: a: simvastatin; b: atorvastatin; c: pravastatin; d: fluvastatin; e: lovastatin; f: rosuvastatin; g: pitavastatin; and h: nonstatin.

As shown in Figure 4, to analyze the consistency of direct evidence and indirect evidence, we divided all studies into closed triangles and calculated the absolute difference expressing as ROR between direct and indirect evidence in each closed loop. For the ROR, there was a 95% confidence interval. When it includes 1, which means direct evidence and indirect evidence are highly consistent. In our study, the ROR of 95% confidence interval in the loops of simvastatin-pravastatin-fluvastatin, simvastatin-pravastatin-lovastatin, and atorvastatin-rosuvastatin-nonstatin did not include 1, which displayed that their consistency tests were inconsistency statistically. For others, consistency for direct evidence and indirect evidence was increasing from top to bottom. That indicated the analysis of different types of statins on the incidence of cancer in diabetic patients. For the comparison of Figure 4, the more the bottom of the comparison, the higher the reliability of the results and closer to the above, the more cautious the analysis needs. However, for the loop of atorvastatin-pravastatin-nonstatin, atorvastatin-pitavastatin-nonstatin, atorvastatin-lovastatin-nonstatin, atorvastatin-fluvastatin-nonstatin, simvastatin-lovastatin-nonstatin, simvastatin-fluvastatin-nonstatin, simvastatin-pravastatin-nonstatin, simvastatin-rosuvastatin-nonstatin, simvastatin-atorvastatin-nonstatin, and simvastatin-pitavastatin-nonstatin, because the values of *p* were bigger than 0.05, there were no statistically significant and it needed further studies to analyze their consistencies. Thus, the consistency of lovastatin-rosuvastatin-nonstatin for direct evidence and indirect evidence was the highest and the conclusions between their comparisons were more reliable.

### 3.5. The Effect of Direct Comparison on the Network Meta-Analysis

**Figure 5:**
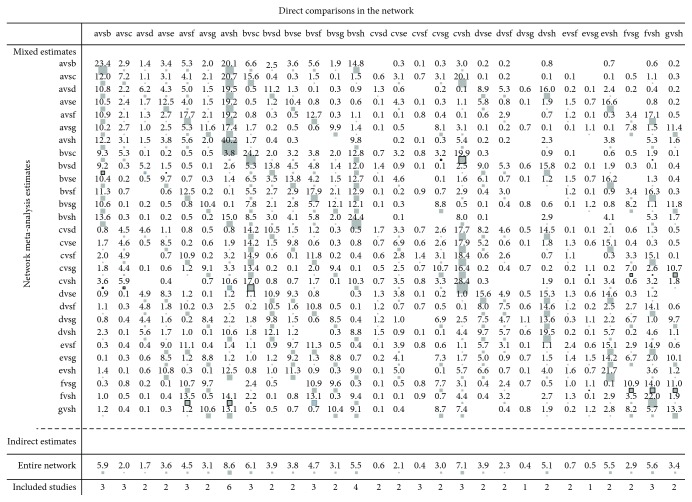
The pairwise comparison contributes to merged comparison. The letters in the figure represented different treatment measures: a: simvastatin; b: atorvastatin; c: pravastatin; d: fluvastatin; e: lovastatin; f: rosuvastatin; g: pitavastatin; and h: nonstatin.

The horizontal axis presents direct comparison, as the vertical axis presents the result of the indirect comparison. The matrix was formed by different comparison in horizontal axis contribution to merged comparison results in vertical axis. From Figure 5, the direct comparison between simvastatin and atorvastatin accounted for 23.4% of the merged comparison of simvastatin and atorvastatin and 5.9% of the entire network. The direct comparison between simvastatin and nonstatin accounted for 40.2% of the merged comparison of simvastatin and nonstatin and 8.6% of the entire network.

### 3.6. The Results of Network Meta-Analysis

**Figure 6:**
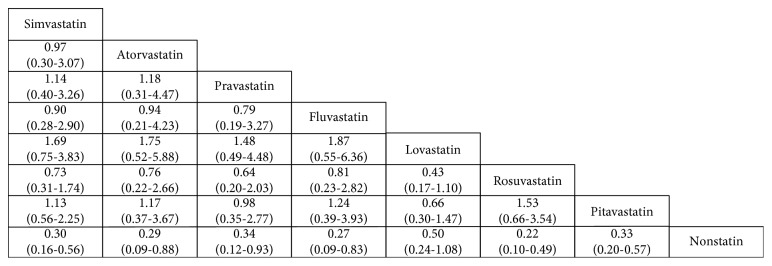
A complete summary of estimates from the network meta-analysis for occurrence of cancer in diabetes patients with statins or not (rate ratios with 95% CI). Statistically significant differences between interventions are shown in bold, underlined font.

Figure 6 shows the network meta-analysis results of the pairwise comparison of the 28 states. For diabetes patients without statins, the usage of simvastatin, atorvastatin, pravastatin, fluvastatin, rosuvastatin, and pitavastatin decreased the occurrence of cancer and their rate ratios with 95% CI were (0.30, 0.16–0.56), (0.29, 0.09–0.88), (0.34, 0.12–0.93), (0.27, 0.09–0.83), (0.22, 0.10–0.49), and (0.33, 0.20–0.57). For the rest, comparisons were not statistically significant (*p* > 0.05).

### 3.7. Ranking of the Incidence of Cancer in Diabetic Patients with Different Types of Statins

**Figure 7:**
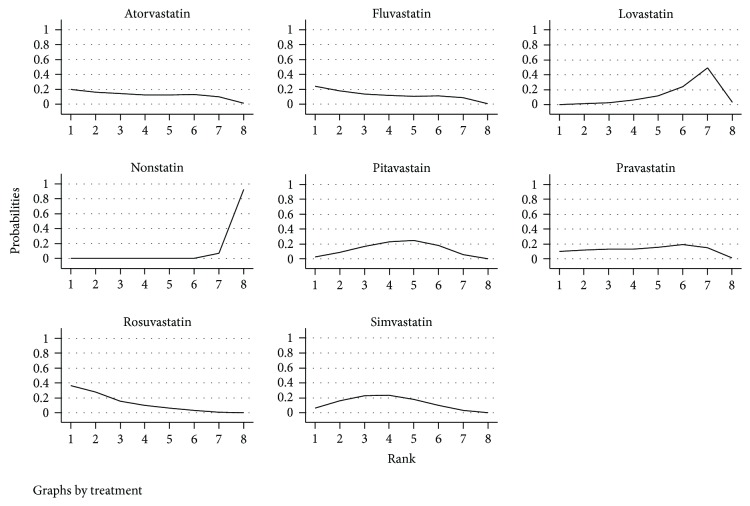
Distribution of probabilities of each intervention is ranked at each of the possible 8 positions. The horizontal axis is the ranking order, and the vertical axis is the probability of a certain order in a certain order.

From Figure 7 and Table 2, the risk of cancer when using statins and nonusing statins in diabetic patients was from the lowest to the highest: rosuvastatin (35.0%), fluvastatin (24.8%), atorvastatin (20.3%), pravastatin (10.4%), simvastatin (6.6%), pitavastatin (2.6%), lovastatin (0.3%), and nonstatin (0.0%). The SUCRA score of 100%, the greater the representative of the treatment may be better. Further, the mean ranks of all interventions in decreasing the occurrence of cancer in diabetes patient were rosuvastatin (2.4), fluvastatin (3.4), atorvastatin (3.7), simvastatin (3.7), pitavastatin (4.3), pravastatin (4.4), lovastatin (6.1), and nonstatin (7.9).

### 3.8. Comparison-Adjusted Funnel Plot

**Figure 8:**
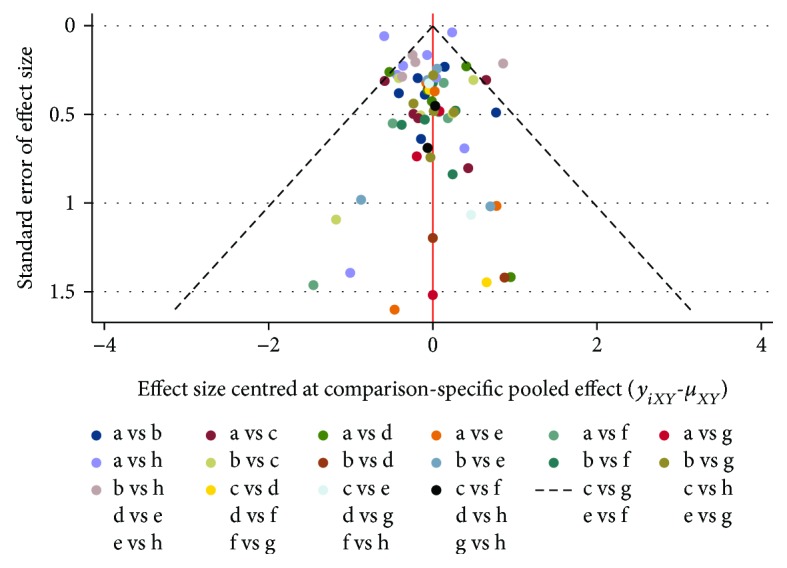
Funnel plots of the meta-analysis. The ordinate (*Y*) presents the effect of each study, and abscissa (*X*) is the total effect on each control group. Scatter represents a study in the comparison of the two treatment measures. The letters in the figure represent different treatment measures: a: simvastatin; b: atorvastatin; c: pravastatin; d: fluvastatin; e: lovastatin; f: rosuvastatin; g: pitavastatin; and h: nonstatin.

The publication bias was observed by plotting the standard error (*Y*) of the total effect of the comparison groups on the funnel pattern of the scatter of the total effect (*X*). 68 scattered points represented 32 studies; 28 different colors represented different comparisons. Most of the scattered points were symmetrically distributed around the vertical line of *X* = 0 (Figure 8), which displayed there was no significant small sample effect or publication bias in the network.

## 4. Discussion

Compared with nondiabetic population, patients with diabetes mellitus, especially T2DM, had a markedly higher incidence of cancer. This relationship was reported in different kinds of cancers, such as breast cancer, liver cancer, lung cancer, gastric cancer, and colorectal cancer [41–45].

There were several explanations for why T2DM could increase the incident rate of cancer. Firstly, patients with T2DM have a high level of insulin in the blood. Hyperinsulinemia might contribute to the proliferation of cancer cells by activating the signaling pathway of IGF-1, which could lead to the occurrence of cancer [46]. Secondly, several studies had indicated that medications which lowered the level of blood glucose might have an effect on the risk of cancer. The risk of cancer may be increased or decreased. When patients were treated with metformin, their risk of cancer may be reduced [47, 48]. However, other drugs had opposed results, including sulfonylureas and thiazolidinedione [49, 50]. Thirdly, hyperglycemia might be another potential reason. There was a systematic review to investigate the relationship between HbA1c and cancer. They found that a higher level of HbA1c was associated with a higher incidence of cancers, including pancreatic, respiratory, and colorectal cancers [51]. Data from individual participant revealed that, after adjusting age, sex, smoking status, and body mass index, diabetes was still a risk factor for cancer death (HR = 1.25 and 95% CI 1.19–1.31) [52]. Besides, T2DM patients with smoking, virus infection, and obesity were more likely to develop a cancer than without [53-55]. However, the effective strategy to reduce the incidence rate of cancer in T2DM patients was limited. Statins may be one of the promising drugs.

In our study, compared with nonstatin, the use of different types of statins in patients with T2DM all appeared to lower risk of cancer. However, more accurate conclusions and ranks in different statins especially for pravastatin, simvastatin, pitavastatin, and lovastatin require more research to verify. A lot of studies have suggested that statins may have a beneficial effect on the risk of cancer. Using the data from the Women's Health Initiative, compared with nonusers, statin user had a significant lower risk of cancer death (HR = 0.78 and 95% = 0.71–0.86) [56]. Among a large cohort of HCV-positive patients, use of statins was reported to reduce the incidence of hepatocellular carcinoma (HCC) [57]. Statin use could also improve survival outcomes in renal cell carcinoma patients with dyslipidemia [58]. However, a recent meta-analysis which included 14 studies showed that statins had no effect on the risk of prostate cancer [59]. According to a nationwide study in Denmark, the incidence rate of squamous cell skin cancer (SCC) was not decreased, although statin use [60]. Therefore, the effect of statin use on the risk of cancer is still controversial.

Nevertheless, there are several limitations which should be mentioned. First, the populations in this study were not the same. Most of them were from the United States. Second, unpublished studies were not included, which may have a difference to the results. Third, because of cancer latency, the incidence of cancer may be underestimated, which may have an effect on the validity of the outcomes. Fourth, several confounders, such as therapies of glycemic control and treatment of cancers, were not analyzed.

In conclusion, our network meta-analysis clearly presents the advantage of statin usage in cancer prevention of patients with T2DM. Rosuvastatin may be the best of all for reducing cancer risk of T2DM patients compared with others. Our current work could provide new insights into the role of statins on cancer prevention. Given the different population using statins, latency for cancer of T2DM patients, and long-term usage of statins, it is necessary to design RCTs well and continue monitoring the safety of statins.

## Figures and Tables

**Table 1:** Characteristics of included studies in this network meta-analysis.

First author	Year	Country/district	Study period	Number of patients	Cancer types	ICD	Research types	Statin types	Quality of scores
Chen [34]	2015	Taiwan	2000–2010	2053	Hepatocellular carcinoma	ICD-9-CN: 155	Case-control study	a, b, c, d, e, f	8/9
El–Serag [35]	2009	United States	1997–2002	3302	Hepatocellular carcinoma	ICD-9-CM: 155	Case-control study	a	8/9
Lee [37]	2012	Korea	1999–2008	1920	Gastric cancer	ICD: 16.0–16.9	Case-control study	a, b, c, f, g	8/9
Beishuizen [36]	2004	Netherlands	2001–2003	182	Malignancies	/	RCT	a	5
Wanner [38]	2005	Germany	1998–2004	1255	Cancer	/	RCT	b	5
Kim [39]	2016	Korea	2002–2013	154	Hepatocellular carcinoma	ICD C22.0	Case-control study	a, b, c, d, e, f, g	8/9
Hachem [40]	2009	United States	1997–2002	14,707	Colorectal cancer	ICD-9: 153.0–153.9154.0–154.1154.8230.3230.4	Case-control study	a	8/9

RCT: randomized controlled trial; /: there is no record of the ICD of cancer; ICD: international classification of disease; a: simvastatin; b: atorvastatin; c: pravastatin; d: fluvastatin; e: lovastatin; f: rosuvastatin, g: pitavastatin.

**Table 2:** Ranking curve (SUCRA) value of per intervention.

Treatment	SUCRA	PrBest	Mean rank
Nonstatin	1.1	0.0	7.9
Simvastatin	61.2	6.6	3.7
Atorvastatin	61.9	20.3	3.7
Pravastatin	51.4	10.4	4.4
Fluvastatin	65.6	24.8	3.4
Lovastatin	26.5	0.3	6.1
Rosuvastatin	80.1	35.0	2.4
Pitavastatin	52.2	2.6	4.3

PrBest: the probability of becoming the best treatment; mean rank: the average ranking of treatment measures; SUCRA: surface under the cumulative.
